# Can geroscience be translated into healthcare?

**DOI:** 10.1007/s00391-024-02326-z

**Published:** 2024-07-09

**Authors:** Luigi Ferrucci, Toshiko Tanaka, M. Cristina Polidori

**Affiliations:** 1 Translational Gerontology Branch, Intramural Research Program, National Institute on Aging, National Institutes of Health, 251 Bayview Boulevard, 21224 Baltimore, MD USA; 2 Department II of Internal Medicine and Center for Molecular Medicine Cologne, Ageing Clinical Research, Cologne, Germany; 3 Cluster of Excellence—Cellular Stress Responses in Aging-Associated Diseases, University of Cologne, Cologne, Germany

**Keywords:** Life span, Biomarker, Phenotype, Entropy, Rate of aging, Lebensspanne, Biomarker, Phänotyp, Entropie, Alterungsgeschwindigkeit

## Abstract

As an introduction to this special issue on geroscience, the present work focuses on the complexity of disentangling biomolecular mechanisms of aging from biopsychosocial causes of accelerated aging. Due to this complexity, the biomolecular aging hallmarks of frailty and multimorbidity as predominant aging phenotypes in geriatrics reflect single aspects of the aging process. A possible approach to facilitate the integration of geroscience into healthcare might be to consider aging as the dynamic ratio between damage accumulation at the molecular and cellular level and resilience as strategies that prevent or repair such damage. There is a large body of evidence to show that geroscience has the potential to change healthcare; however, reaching a consensus and translating the best tool to measure aging needs more research on 1) the sensitivity of biomarkers to interventions and 2) the relationship between changes in biomarkers and changes in health trajectories.

## Abstract (de)

Als Einleitung zu diesem Sonderheft über Geroscience konzentriert sich die vorliegende Arbeit auf die Komplexität der Entflechtung biomolekularer Mechanismen des Alterns von biopsychosozialen Ursachen des beschleunigten Alterns. Aufgrund dieser Komplexität spiegeln sowohl die biomolekularen Alterungsmerkmale einerseits als auch Frailty und Multimorbidität als vorherrschende Alterungsphänotypen in der Geriatrie andererseits einzelne Aspekte des Alterungsprozesses wider. Ein möglicher Ansatz zur Erleichterung der Integration der Geroscience in die Gesundheitssysteme könnte darin bestehen, das Altern als dynamisches Verhältnis zwischen der Akkumulation von Schäden auf molekularer und zellulärer Ebene und der Resilienz als Strategien zur Verhinderung oder Reparatur solcher Schäden zu betrachten. Es gibt zahlreiche Evidenz dafür, dass Geroscience das Potenzial hat, die Gesundheitssysteme zu verändern. Um jedoch einen Konsens zu erzielen und das beste Instrument zur Messung des Alterns zu finden, bedarf es weiterer Forschung zu 1) der Sensitivität von Biomarkern gegenüber Interventionen und 2) dem Zusammenhang zwischen Veränderungen bei Biomarkern und Gesundheitsverlauf.

The global transformation of the demographic structure of the population with rapid expansion of old persons, and especially of the oldest old, makes geriatric care the highest priority. As advances in medicine and public health succeed in achieving longer life spans, the focus has shifted to expanding health span and maximizing the quality of life. To achieve this goal, there has been a reconceptualization of the view of aging in the research community. The geroscience hypothesis poses that the biological mechanisms that drive aging are the root cause of the phenotypic transformations that are typically observed with aging and their functional consequences, such as loss of mobility and cognition [16]. There is mounting evidence that the life span can be expanded and some aspects of aging can be slowed down in preclinical models and generated great enthusiasm for the possibility of human applications and considerable investments from both the private and public sectors. The global anti-aging industry is estimated at US $ 71.6 billion in 2023, and is expected to reach US $120.4 billion by 2032, reflecting the demand for translation of the geroscience hypothesis to humans [1].

The geroscience hypothesis is supported by a multitude of preclinical studies suggesting that aging is malleable and that slowing down aging results in the expansion of both longevity and health span [9]; however, the idea that the pace of aging can be modulated through interventions is not universally accepted and certainly not a mainstream concept in the medical field; however, aging has become a popular topic of conversation at social occasions and in the popular press with different aspects of aging being highlighted in the media almost every week [7, 19]. Due to the complexity and multifactorial nature, aging is not easy to operationalize for scientific purposes beyond the mere counting of days after birth. There is a progressive decline of health with passage of time but there is heterogeneity in the aging process. Some people undergo gradual changes with age, while others experience a rapid decline. This leads to greater variability in phenotypes, particularly among older individuals, where people of the same chronological age can exhibit a vastly different health status. Multimorbidity or frailty have been used to capture such heterogeneity but it is evident that these concepts fail to comprehensively describe the experience of becoming old, sick, and impaired. To make progress, a universal concept of aging should be available that can be operationalized and quantified. Armed with such conceptualization, we could start testing the geroscience hypothesis and, hopefully translate it into community health and medicine.

A possible approach is to consider aging as the dynamic ratio between damage accumulation at the molecular and cellular level and resilience strategies that prevent or repair such damage [2]. As all physical entities, macromolecules are subject to entropy defined as a gradual increase in the degree of disorder. The concept is best explained by examples. Native, mature proteins have specific amino acid sequences, folding and aggregation in units that enable them to optimally perform their biological activity. Over time, all proteins as physical objects are subject to entropy and undergo damage, some amino acids may be chemically damaged, and the 3D structure altered to the point that the biological function is diminished or lost. A state of order has been disrupted and some random alternations have increased disorder, ergo the entropy in this protein has increased. Several biological mechanisms have been evolutionary selected to preserve the function of proteins as well as other molecules. Misfolded proteins can be refolded by a complex stress-response system that detect them and activate chaperon proteins that can refold them [5]. If the proteins cannot be refolded, they can be tagged for elimination through chaperon-mediated autophagy and proteasomal degradation, followed by the synthesis of new fully functional proteins. There is no relevant damage accumulation while these systems work, and operationally there is no aging. Incidentally, this model of aging is consistent with Schrödinger’s view that “life is a low entropy state” and “aging happens as entropy increases” [17]. It is worth mentioning that maintaining the state of low entropy requires substantial amounts of energy, suggesting that conditions of low energy availability may lead to accelerated aging.

The idea of geroscience is relatively simple and the potentials for benefits are gigantic: if aging is driven by the entropic accumulation of damage that is counteracted by resilience mechanisms that prevent damage accumulation or repair it, then enhancing these resilience mechanisms can potentially promote health over the life span. Key consequences of these hypothesis are slowing aging can be started in early life when “aging” pathologies are accumulating but no detectable evidence of clinical disease or impairment is present. The mechanisms of molecular and cellular resilience should be essentially similar in all cells and, therefore, empowering these tolls of “intrinsic” prevention should effectively prevent or delay most late life pathologies. This would be a tremendous achievement at a time when the prevalence of morbidity and multimorbidity is globally increasing due to extended life span. The ability to reduce the period of life characterized by diseases and disability and adding years spent in good health is an attractive opportunity, and perhaps it is also the only chance to accomplish the economic sustainability of healthcare systems even in the wealthier nations.

Let us imagine that some interventions exist that slow the aging process and have shown none or minimal side effect in a phase I human trial. How can we demonstrate that these interventions slow down aging in humans? Functional outcomes such as frailty or disability, or even death would require long follow-up times and are not feasible financially. A possible solution to this conundrum is the development of biological aging biomarkers, or more precisely, biomarkers of the level of damage accumulation due to aging. If aging is causing diseases and disability, slowing down aging should do the trick, and in fact this is one of the central questions of aging research. Recent advances in the discovery of aging biomarkers have been based on molecules measured in biological fluids, typically called omics. A narrative review of the work in this field is outside the scope of this editorial; here it would be sufficient to say that many of the omics indices of age correlate surprisingly well with chronological age and the deviations between the omics age with chronological aging predict important adverse health outcomes, including chronic diseases, frailty and mortality [4, 8, 12, 20]. The robustness and predictive validity of these tools for the measurement of global and specific health dimensions is improving every day, and large projects are currently ongoing that should enable making further progress. Would the availability of these tools be enough for the clinical translation of the geroscience hypothesis?

We will start by dreaming about an ideal implementation. Imagine that every person seeking medical care for a specific problem could also be assessed by one or more composite aging biomarkers. Beyond the diagnosis and treatment of the emerging medical problem, all patients could receive an evaluation of the pace of aging, for the whole body and for organ systems. This evaluation would call for tailored preventative interventions based on a specific biomarker that could be behavioral, pharmacological or both. Hopefully, individuals assessed with this method and treated by a tailored gerotherapeutic intervention would experience a longer and healthier life. Over time, these data could be integrated into an electronic medical record database and the effectiveness of the intervention tracked, with the possibility of improving the diagnosis and indications over time, perhaps by using AI.

Now we will play devil’s advocates and make predictions on what can go wrong. There are already hundreds of predictive scores for adverse health events published in the literature, including cardiovascular events, falls, disability, frailty, complications of surgery or chemotherapy. Each one of these “risk scores” was validated in one or multiple population with a methodology rigorous enough to deserve publication in peer review journals, and only a minuscule fraction of them is used in clinical practice. Geroscience is a beautiful and powerful idea but the “bedside to bookcase” trap is lingering in a corner! Many gerontologists and geriatricians have spent many perfectly good hours or even days debating the best definition of frailty, typically with no final agreement and without coming up with a strong, convincing argument that frailty should be widely assessed in older patients. Indeed, it has been suggested that unless we can demonstrate that making a diagnosis of frailty changes the therapeutic approach and, especially some relevant clinical outcome, it will be difficult to convince clinicians that assessing frailty is worth their time. Similarly, while a handful of studies are beginning to show that the aging clocks change over time [11] and are responsive to interventions [6], it remains unclear whether these biomarkers can guide clinicians in determining therapeutic strategies or are predictive of subsequent health trajectories. It is a high bar but unavoidable to achieve translation of results from cutting edge research to the clinic or to public health.

It would be best to avoid long theoretical discussions and make sure that our ideas generate actions. Geroscience has the potential to change health care; however, reaching a consensus and translating the best tool to measure aging will take time. A more grassroots approach, in which informed geriatricians apply the tools with the best evidence and share their experiences with other clinicians, could expedite these efforts. With a strong effort towards translation, the application of geroscience concepts has the potential to become a powerful weapon in the hands of clinicians to combat the rising burden of pathology in an increasingly aging population. A couple of additional roadblocks deserve to be mentioned. First, efforts to slow the pace of aging by reducing the burden of damage and/or enhance biological resilience strategies will be most effective when a degree of residual resilience and biological integrity still exists. Ergo, the people who can benefit the most from gerotherapeutics are those who have no clinical evidence of a disease; however, even if we could demonstrate that such interventions slow the pace of aging measured by biomarkers, there is no guarantee that this will result in health span extension and/or that long-term side effects will overcome the possible short-term benefits. Without such robust evidence, it is hard to propose to start a treatment in people who are apparently healthy and by improvements in biomarker profiles would result in positive effects on health in a decade. Indeed, physicians would be hesitant to prescribe such interventions. Unavoidably, the initial trials will have to be done in patients who show some level of impairment, such as those affected by mild diseases or those who are at high risk of developing serious medical conditions.

The second issue concerns the meaning of biomarkers. Molecules that can be detected in body fluids may not be reflective of what is happening at the tissue level. Not only do we not know where they come from, we also do not know what they mean in terms of the underlying biology and pathology. Let us take the example of protein biomarkers, at the current state of knowledge, we can classify circulating biomarkers into 4 broad categories:

1. Proteins that are leaking from specific organs and indicate organ specific damage. This type of biomarker is familiar to physicians. For example, rising levels of blood troponin are routinely used for the early diagnosis of myocardial infarction. Recent data demonstrated that subclinical diseases of specific organs are associated with rising levels of organ-specific proteins [13]. While this concept is already close to what is used in the clinical field, a comprehensive characterization of organ-specific proteins or protein profiles in research is in the early stages. The development of blood tests that can detect splicing variants of the same protein may further increase the specificity of these assays, as there are many examples of protein-splicing variants selectively produced by different organs [10].
2. Proteins that provide clues on the biology of aging mechanisms that may be causative of damage accumulation and the emergence of pathology. Note that because of the operational definition used here, these biomarkers could indicate damage accumulation but may also be indicative of exhaustion of resilience mechanisms.
3. Proteins that mark the activation of resilience mechanisms that oppose damage accumulation. It is important to note that biomarkers in categories 2 and 3 are very difficult to distinguish. They both increase with aging, and naturally their levels are correlated and predictive of accumulation of pathologies. Such a distinction is important because whereas proteins in category 2 would be promoting age acceleration and could be a target for geroprotective therapeutics, proteins in category 3 are compensatory and their underlying biological mechanisms should be enhanced. There have been attempts to distinguish them by Mendelian randomization in large databases, but the results are not fully convincing yet and have not been extensively replicated [3]. For example, genetic predictors of growth differentiation factor 15 (GDF-15) levels are not strongly associated with mortality, although GDF-15 is the circulating protein most strongly associated with age and one of the most powerful predictors of multiple chronic diseases.
4. Last but not least, there are proteins that show fluctuations with aging but, independent of chronological age, are not predictors of important outcomes. While these proteins may not be considered biomarkers of the aging process, they represent opportunities for future research work. The simple classification reported here is clearly an oversimplification and as our understanding of the meaning of biomarkers improves, different logical groupings may emerge. In addition, while these examples address proteins, molecular features that track damage accumulation are diverse, and include (but are not limited to) metabolites, epigenetic markers, transcriptomics, proteomics, cell-free mitochondrial DNA and many others.

A discussion of aging cannot be complete without touching on inflammation. There is overwhelming evidence that inflammation is a common factor for all major chronic diseases. In the CANTOS trial, canakinumab therapy blocking the interleukin‑1 beta innate immunity significantly reduced the rate of recurrent cardiovascular events compared to placebo [15]. Reanalysis of the CANTOS data shows that anti-inflammatory treatment also reduces the rates of lung cancer, severe diabetes, hip fracture, congestive heart failure and anemia although not frailty [14, 18]. While inflammation has been considered one of the hallmarks of aging, the study of the molecular mechanisms of inflammation tells a different story. As mentioned above, many of the hallmarks can be considered resilience mechanisms and their dysfunction is permissive of damage accumulation with aging. Although the mechanisms by which the damaged molecules become immunoreactive are likely many, overall damage calls for an inflammatory response aimed to clear the system of pieces of broken machinery. In this sense, inflammation is reflective of the burden of molecular damage. Likely, once inflammation is activated above a certain threshold, it initiates a vicious cycle that accelerates damage accumulation. Thus, the key is to regulate inflammation in a manner that retains its beneficial functions while preventing the harmful effects of an exaggerated response. We are not yet able to strike this intricate balance.

Recently, the concept of resilience has been assimilated with the Greek and Roman philosophy of stoicism. Conceptually, stoicism is the ability to be resilient in the face of setbacks and disasters. There is a lot of stoicism in our body. The human body has been much celebrated in poetry, but its biological complexity may be intimidating. This could be why its potentials for self-healing have been taken for granted and not considered for the development of therapeutic targets. Enhancing stoicism and monitoring its impact using biomarkers could represent the breakthrough in medicine that we’ve been anticipating. Research on this topic is a priority for human health and should be prioritized at all levels.

## Declarations

This article does not contain any studies with human participants or animals performed by any of the authors.
